# SARS-CoV-2 Spike Proteins and Cell–Cell Communication Inhibits TFPI and Induces Thrombogenic Factors in Human Lung Microvascular Endothelial Cells and Neutrophils: Implications for COVID-19 Coagulopathy Pathogenesis

**DOI:** 10.3390/ijms231810436

**Published:** 2022-09-09

**Authors:** Biju Bhargavan, Georgette D. Kanmogne

**Affiliations:** Department of Pharmacology and Experimental Neuroscience, College of Medicine, University of Nebraska Medical Center, Omaha, NE 68198-5800, USA

**Keywords:** SARS-CoV-2 spike proteins, human lung endothelial cells, neutrophils, tissue factor, TFPI, DTNB, Factor-V, thrombin, thrombomodulin, fibrinogen

## Abstract

In SARS-CoV-2-infected humans, disease progression is often associated with acute respiratory distress syndrome involving severe lung injury, coagulopathy, and thrombosis of the alveolar capillaries. The pathogenesis of these pulmonary complications in COVID-19 patients has not been elucidated. Autopsy study of these patients showed SARS-CoV-2 virions in pulmonary vessels and sequestrated leukocytes infiltrates associated with endotheliopathy and microvascular thrombosis. Since SARS-CoV-2 enters and infects target cells by binding its spike (S) protein to cellular angiotensin-converting enzyme 2 (ACE2), and there is evidence that vascular endothelial cells and neutrophils express ACE2, we investigated the effect of S-proteins and cell–cell communication on primary human lung microvascular endothelial cells (HLMEC) and neutrophils expression of thrombogenic factors and the potential mechanisms. Using S-proteins of two different SARS-CoV-2 variants (Wuhan and Delta), we demonstrate that exposure of HLMEC or neutrophils to S-proteins, co-culture of HLMEC exposed to S-proteins with non-exposed neutrophils, or co-culture of neutrophils exposed to S-proteins with non-exposed HLMEC induced transcriptional upregulation of tissue factor (TF), significantly increased the expression and secretion of factor (F)-V, thrombin, and fibrinogen and inhibited tissue factor pathway inhibitor (TFPI), the primary regulator of the extrinsic pathway of blood coagulation, in both cell types. Recombinant (r)TFPI and a thiol blocker (5,5′-dithio-bis-(2-nitrobenzoic acid)) prevented S-protein-induced expression and secretion of Factor-V, thrombin, and fibrinogen. Thrombomodulin blocked S-protein-induced expression and secretion of fibrinogen but had no effect on S-protein-induced expression of Factor-V or thrombin. These results suggests that following SARS-CoV-2 contact with the pulmonary endothelium or neutrophils and endothelial–neutrophil interactions, viral S-proteins induce coagulopathy via the TF pathway and mechanisms involving functional thiol groups. These findings suggest that using rTFPI and/or thiol-based drugs could be a viable therapeutic strategy against SARS-CoV-2-induced coagulopathy and thrombosis.

## 1. Introduction

Severe acute respiratory syndrome coronavirus-2 (SARS-CoV-2), the causative agent of coronavirus disease 2019 (COVID-19), is a novel betacoronavirus that was first reported in China in December 2019 and quickly spread across the globe [1,2]. It has, so far, infected over 601 million people worldwide, resulting in over 6.47 million deaths and counting [3,4,5]. Postmortem examination shows that, for most of these individuals, the primary cause of death was acute respiratory distress syndrome involving severe lung injury, which was often associated with a cytokine storm, coagulopathy, and thrombosis of the alveolar capillaries [6,7,8,9]. Autopsy and biopsy study of COVID-19 patients also demonstrated the presence of SARS-CoV-2 virions and spike (S) proteins in pulmonary vessels and skin lesions as well as sequestrated leukocytes infiltrates associated with endotheliopathy, increases in circulating prothrombotic factors, microvascular injury, and thrombosis [7,8,10,11,12]. The reasons for these pulmonary complications in COVID-19 patients are unknown.

Like other coronaviruses, SARS-CoV-2 enters and infects target cells by binding its S-protein to cellular angiotensin-converting enzyme 2 (ACE2), followed by priming by the transmembrane serine protease-2 [13,14,15]. S-protein is a transmembrane glycoprotein; its S1 subunit contains the viral receptor binding domain (RBD) that specifically recognizes the ACE2 protease domain (PD) on target cells. RBD–PD interaction is critical for viral binding to cellular ACE2, entry, and infection of target cells [14,15]. There is evidence that neutrophils [16,17] and endothelial cells from different vascular beds [18,19,20] express ACE2. There is also evidence that viral S-proteins can be shed; are present in bodily fluids, microvessels, and tissues of SARS-CoV-2 infected patients; and can directly affect the vascular endothelium [12,21,22,23,24]. Evidence also suggests that long COVID-19 syndrome involves persistent microvascular endotheliopathy associated with increased SARS-CoV-2 virions and S-proteins in the blood and tissues as well as increased peripheral levels of pro-thrombotic factors [25,26,27]. In the present study, we investigate the direct and indirect effects of S-proteins’ exposure on expression and secretion of thrombogenic factors by primary human lung microvascular endothelial cells (HLMEC) and neutrophils. We demonstrate that exposure of primary HLMEC or neutrophils to S-proteins and endothelial–neutrophil interactions induced transcriptional upregulation of tissue factor (TF), with significantly higher TF levels observed with S-proteins of the SARS-CoV-2 Delta variant, compared to the Wuhan variant S-proteins. Cell exposure to S-proteins and endothelial–neutrophil interactions significantly increased the expression and secretion of Factor-V, thrombin, and fibrinogen, all of which are clotting and pro-thrombotic factors [28,29,30]. S-proteins also inhibited tissue factor pathway inhibitor (TFPI), the primary physiological inhibitor of the extrinsic pathway of blood coagulation [31,32,33,34]. Recombinant TFPI (rTFPI), as well as the thiol blocker 5,5′-dithio-bis-(2-nitrobenzoic acid) (DTNB) blocked S-protein-induced expression and secretion of Factor-V, thrombin, and fibrinogen. Thrombomodulin (TM), an endothelial receptor that binds thrombin to induce fibrinolysis and prevent/reduce coagulation [35,36], blocked S-protein-induced expression and secretion of fibrinogen but had no effect on S-protein-induced expression and secretion of Factor-V or thrombin. These results suggest that following direct contact of SARS-CoV-2 with the pulmonary endothelium or neutrophils, viral S-proteins induce coagulopathy via the TF pathway and mechanisms involving functional thiol groups. Furthermore, any of these two cell populations exposed to SARS-CoV-2 or viral S-proteins can induce coagulopathy in non-exposed neighboring cells. These findings suggest that viable therapeutic strategy against SARS-CoV-2-induced coagulopathy and thrombosis could include rTFPI and/or thiol-based drugs.

## 2. Results

### 2.1. Exposure of HLMEC and Neutrophils to S-Proteins and Endothelial–Neutrophil Interactions Induced TF (Factor-III) Transcriptional Upregulation

**Figure 1:**
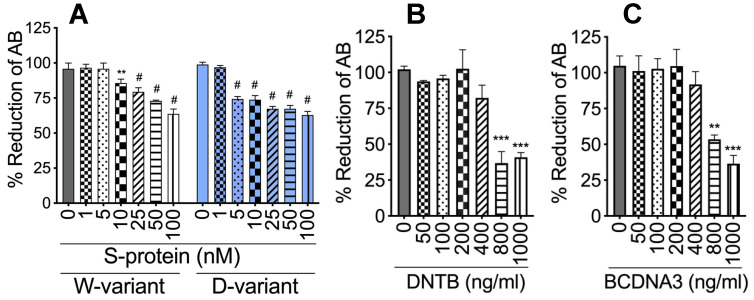
Cytotoxicity of S-proteins, rTFPI, and DTNB on HLMEC. HLMEC were treated with S-proteins (**A**), rTFPI (**B**), and DTNB (**C**) and cytotoxicity at 24 to 48 h assessed by AlamarBlue assay. ** *p* < 0.004; *** *p* < 0.0007; # *p* < 0.0001, compared to untreated controls.

**Figure 2:**
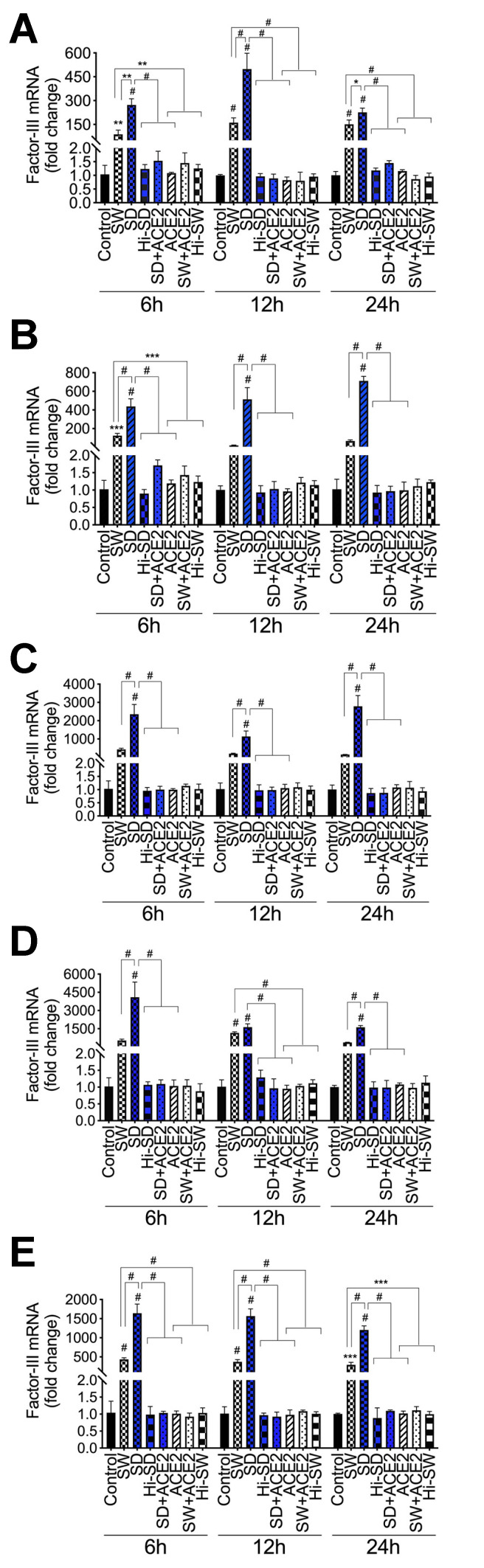
S-proteins and endothelial–neutrophil interactions induce Factor-III transcriptional upregulation in HLMEC and neutrophils. HLMEC were treated with 1 nM S-protein Wuhan (SW) or Delta (SD) variants for 6–24 h (**A**). In separate experiments, HLMEC were treated with S-proteins for 6 h, washed, and co-cultured with neutrophils for 6–24 h (**B**,**C**); neutrophils were treated with S-proteins for 6 h, washed, and co-cultured with HLMEC for 6–24 h (**D**,**E**). Each cell type was harvested separately and Factor-III mRNA levels in endothelial cells (**A**,**B**,**D**) and neutrophils (**C**,**E**) were quantified by real-time PCR. Data presented as mean ± standard deviation. Control: untreated cells; ACE2: cells treated with recombinant human ACE2 (1 µg/mL). Hi: cells treated with heat-inactivated S-proteins. * *p* = 0.01; ** *p* = 0.002; *** *p* < 0.0008; # *p* < 0.0001.

Using S-proteins of two different SARS-CoV-2 variants (Wuhan (SW) and Delta (SD) variants), dose-dependent experiments showed that 48 h treatment of HLMEC with SW (1–5 nM) or SD (1 nM) did not induce toxicity (Figure 1). Therefore, 1 nM S-proteins were used for all subsequent experiments. Exposure of HLMEC to SW for 6 h, 12 h, and 24 h increased TF mRNA, respectively, by 84-fold (*p* = 0.002), 161-fold (*p* < 0.0001), and 149-fold (*p* < 0.0001) compared to untreated cells and cells treated with heat-inactivated (Hi) SW (Figure 2A). Exposure of HLMEC to SD for 6 h, 12 h, and 24 h increased TF mRNA respectively, by 263-fold (*p* < 0.0001), 498-fold (*p* < 0.0001), and 224-fold (*p* < 0.0001) compared to untreated cells and cells treated with Hi-SD (Figure 2A). Co-culture of SW-treated HLMEC with neutrophils increased TF mRNA in HLMEC by 19- to 121-fold (Figure 2B) and increased TF mRNA in neutrophils by 144- to 418-fold (Figure 2C). Co-culture of SD-treated HLMEC with neutrophils increased TF mRNA in HLMEC by 417- to 694-fold (Figure 2B) and increased TF mRNA in neutrophils by 1118- to 2761-fold (Figure 2C). Co-culture of SW-treated neutrophils with HLMEC increased TF mRNA in HLMEC by 364- to 1141-fold (Figure 2D) and increased TF mRNA in neutrophils by 293- to 419-fold (Figure 2E). Co-culture of SD-treated neutrophils with HLMEC increased TF mRNA in HLMEC by 1617- to 4012-fold (Figure 2D) and increased TF mRNA in neutrophils by 1204- to 1583-fold (Figure 2E). 

### 2.2. Delta-Variant S-Proteins Induced Higher TF Levels Compared to Wuhan-Variant S-Proteins

TF levels in SD-treated HLMEC were 1.5- to 3-fold higher than levels in SW-treated HLMEC (Figure 2A). In HLMEC treated with S-proteins and co-cultured with neutrophils, SD induced 3.5- to 27-fold higher TF in HLMEC (Figure 2B) and 5.45- to 19-fold higher TF in neutrophils (Figure 2C), compared to SW. In neutrophils treated with S-proteins and co-cultured with HLMEC, SD induced 1.4- to 7.6-fold higher TF in HLMEC (Figure 2D) and 3.78- to 4.23-fold higher TF in neutrophils (Figure 2E), compared to SW. No significant increase in TF was observed in cells treated with Hi-SW or Hi-SD; recombinant human ACE2 (rhACE2) blocked or significantly abrogated SW- and SD-induced TF (Figure 2).

### 2.3. Exposure of HLMEC and Neutrophils to S-Proteins and Endothelial–Neutrophil Interactions Increased the Expression and Secretion of Factor-V and Thrombin

In the coagulation cascade extrinsic pathway, activated Factor-V catalyzes thrombin generation (catalyzes the conversion of prothrombin to thrombin) [28,29,30]. Therefore, we assessed the effects of S-proteins and endothelial–neutrophil interactions on Factor-V (activated and non-activated) and thrombin expression and secretion.

**Figure 3:**
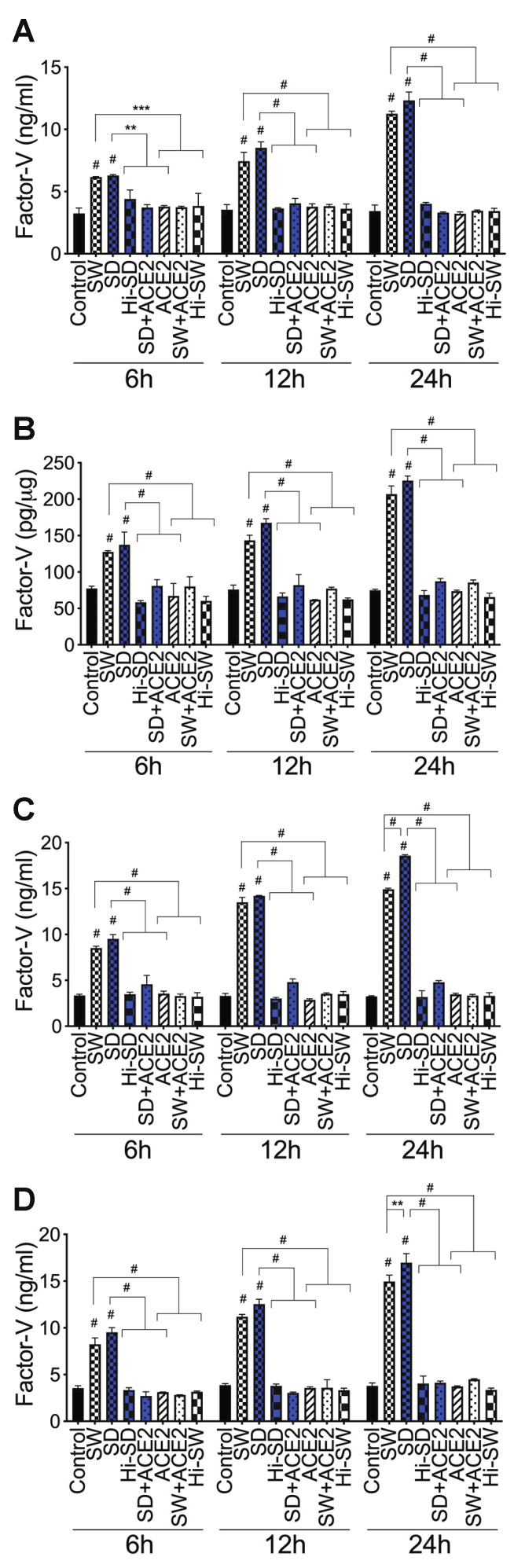
S-proteins and endothelial–neutrophil interactions increase Factor-V expression and release in HLMEC. (**A**,**B**) HLMEC treated with S-proteins (1 nM SW or SD) for 6–24 h; (**C**) HLMEC treated 6 h with S-proteins, washed, and co-cultured with neutrophils for 6–24 h; (**D**) neutrophils treated 6 h with S-proteins, washed, and co-cultured with HLMEC for 6–24 h. Following treatments, Factor-V levels in culture supernatants (**A**,**C**,**D**) and endothelial cell lysates (**B**) were quantified by ELISA. Data presented as mean ± standard deviation. Control: untreated cells; Hi-SW: cells treated with 1 nM heat-inactivated SW; Hi-SD: cells treated with 1nM heat-inactivated SD; ACE2: cells treated with rhACE2 (1 µg/mL). ** *p* = 0.002; *** *p* < 0.0002; # *p* < 0.0001.

***Factor-V:*** Compared to untreated cells, cells treated with Hi-SW, cells treated with Hi-SD, or cells pretreated with rhACE2, exposure of HLMEC to SW or SD for 6 h, 12 h, and 24 h increased Factor-V levels in culture supernatants, respectively, by 1.4- to 1.9-fold, 1.94- to 2.4-fold, and 3- to 3.8-fold (Figure 3A) and increased Factor-V levels in cell lysates by 1.6- to 3.3-fold (Figure 3B). Co-culture of SW- or SD-treated HLMEC with neutrophils for 6 h, 12 h, and 24 h increased Factor-V levels in culture supernatants, respectively, by 2- to 2.8-fold, 3- to 4.95-fold, and 3.87- to 5.8-fold (Figure 3C). Co-culture of SW- or SD-treated neutrophils with HLMEC for 6 h, 12 h, and 24 h increased Factor-V levels in culture supernatants, respectively, by 2.3- to 3.5-fold, 2.9- to 4-fold, and 3.3- to 4.56-fold (Figure 3D). rhACE2 blocked SW- and SD-induced Factor-V expression and secretion. Factor-V levels in cells treated with Hi-SW, Hi-SD, or rhACE2 were similar to levels in untreated controls (Figure 3).

**Figure 4:**
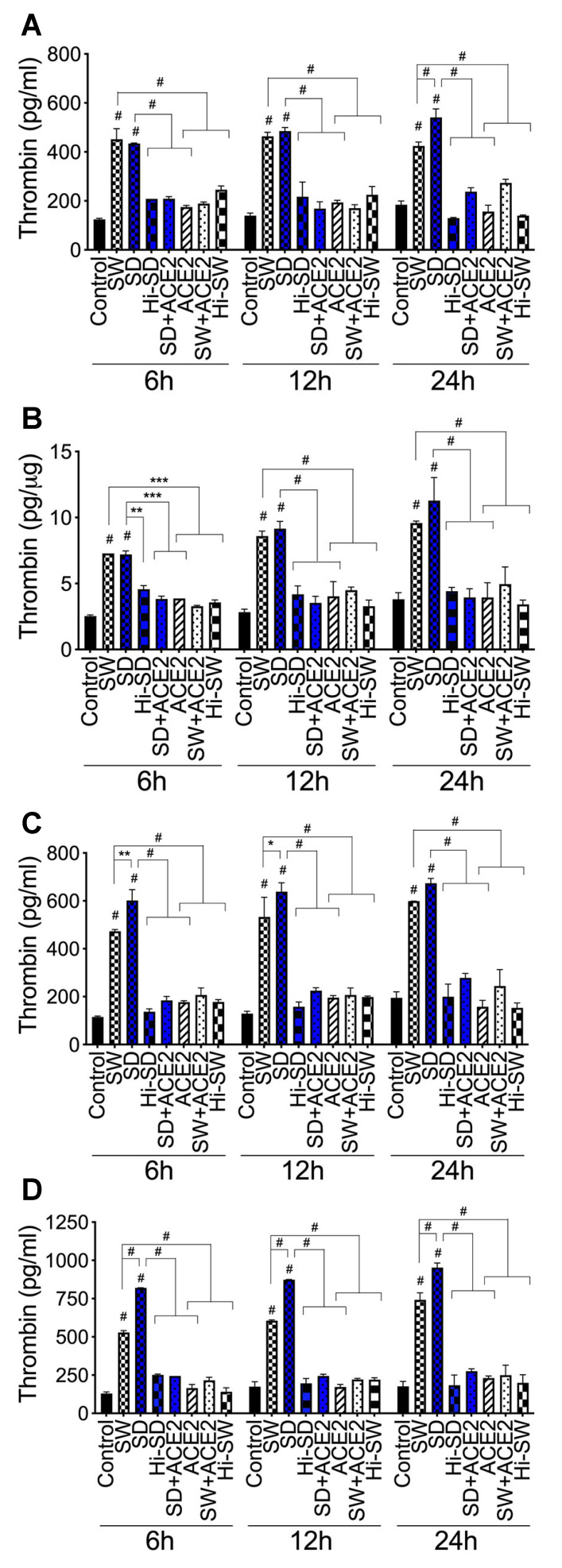
S-proteins and endothelial–neutrophil interactions induce thrombin expression and release in HLMEC. (**A**,**B**) HLMEC treated with S-proteins (1 nM SW or SD) for 6–24 h; (**C**) HLMEC treated 6 h with S-proteins, washed, and co-cultured with neutrophils for 6–24 h; (**D**) neutrophils treated 6 h with S-proteins, washed, and co-cultured with HLMEC for 6–24 h. Following treatments, thrombin levels in culture supernatants (**A**,**C**,**D**) and endothelial cell lysates (**B**) were quantified by ELISA. Data presented as mean ± standard deviation. Control: untreated cells; Hi-SW: cells treated with 1 nM heat-inactivated SW; Hi-SD: cells treated with 1nM heat-inactivated SD; ACE2: cells treated with rhACE2 (1 µg/mL). * *p* < 0.05; ** *p* < 0.008; *** *p* < 0.0006; # *p* < 0.0001.

***Thrombin:*** Compared to untreated cells, cells treated with Hi-SW, cells treated with Hi-SD, or cells pretreated with rhACE2, exposure of HLMEC to SW or SD for 6–24 h increased thrombin levels in culture supernatants by 1.5- to 4-fold (Figure 4A) and increased thrombin levels in cell lysates by 1.6- to 3.2-fold (Figure 4B). Co-culture of SW- or SD-treated HLMEC with neutrophils for 6–24 h increased thrombin levels in culture supernatants by 2.3- to 5.2-fold (Figure 4C). Co-culture of SW- or SD-treated neutrophils with HLMEC for 6–24 h increased thrombin levels in culture supernatants by 2.45- to 6.3-fold (Figure 4D). SD appears to induce higher thrombin levels than SW (Figure 4). rhACE2 blocked SW- and SD-induced thrombin expression and secretion; thrombin levels in cells treated with Hi-SW, treated with Hi-SD, or pretreated with rhACE2 were similar to levels in untreated controls (Figure 4). 

### 2.4. Exposure of HLMEC and Neutrophils to S-Proteins and Endothelial–Neutrophil Interactions Increased the Expression and Secretion of Fibrinogen 

**Figure 5:**
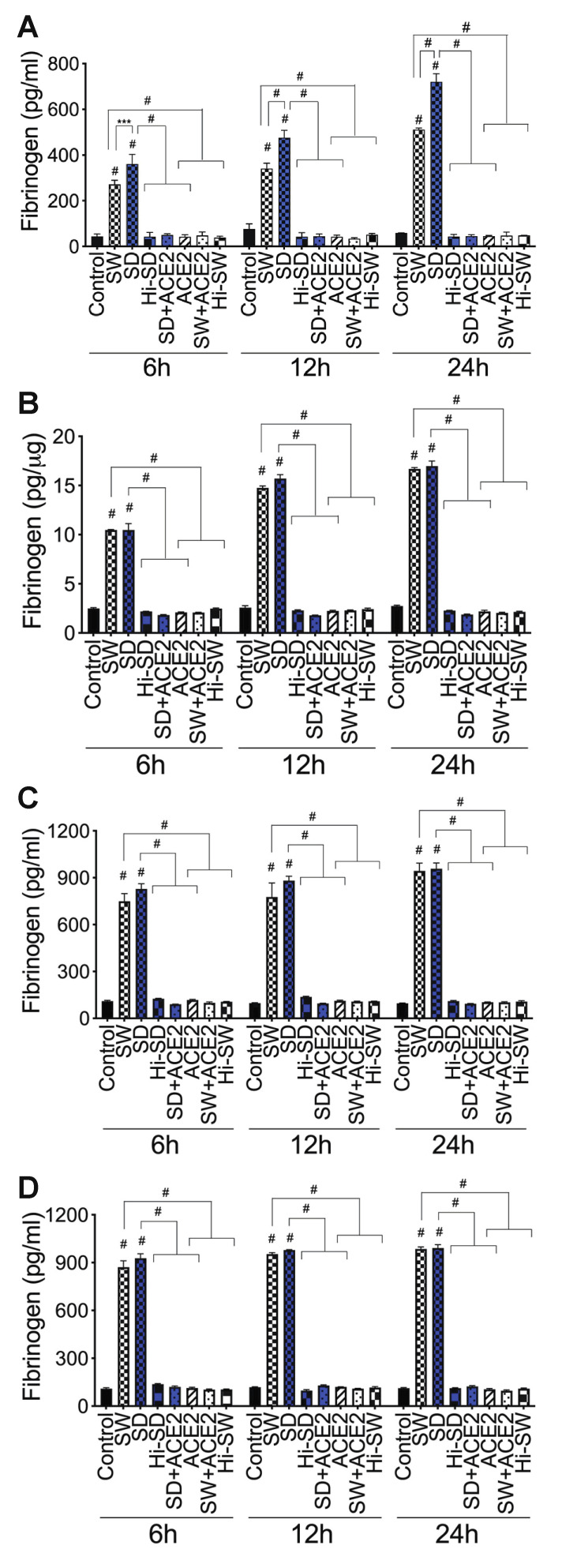
S-proteins and endothelial–neutrophil interactions induce fibrinogen expression and release in HLMEC. (**A**,**B**) HLMEC treated with S-proteins (1 nM SW or SD) for 6–24 h; (**C**) HLMEC treated 6 h with S-proteins, washed, and co-cultured with neutrophils for 6–24 h; (**D**) neutrophils treated 6 h with S-proteins, washed, and co-cultured with HLMEC for 6–24 h. Following treatments, fibrinogen levels in culture supernatants (**A**,**C**,**D**) and endothelial cell lysates (**B**) were quantified by ELISA. Data presented as mean ± standard deviation. Control: untreated cells; Hi-SW: cells treated with 1 nM heat-inactivated SW; Hi-SD: cells treated with 1nM heat-inactivated SD; ACE2: cells treated with rhACE2 (1 µg/mL). *** *p* = 0.0006; # *p* < 0.0001.

In the coagulation cascade, thrombin catalyzes the conversion of fibrinogen to fibrin fibers and blood clots [37,38]. We demonstrate that exposure of HLMEC or neutrophils to S-proteins and endothelial–neutrophil interactions significantly increased fibrinogen expression and secretion. Compared to untreated cells, cells treated with Hi-SW, cells treated with Hi-SD, or cells pretreated with rhACE2, exposure of HLMEC to SW or SD for 6 h, 12 h, and 24 h increased fibrinogen levels in culture supernatants, respectively, by 5.6- to 8.47-fold, 4.4- to 11.25-fold, and 8.7- to 16.4-fold (Figure 5A). Exposure also increased fibrinogen levels in cell lysates by 4.2- to 5.74-fold, 5.7- to 8.78-fold, and 6- to 9-fold (Figure 5B). Co-culture of SW- or SD-treated HLMEC with neutrophils for 6–24 h increased fibrinogen levels in culture supernatants by 6.4- to 10.2-fold (Figure 5C). Co-culture of SW- or SD-treated neutrophils with HLMEC for 6–24 h increased fibrinogen levels in culture supernatants by 6.76- to 10.2-fold (Figure 5D). rhACE2 blocked SW- and SD-induced fibrinogen expression and secretion; fibrinogen levels in cells treated with Hi-SW, Hi-SD, or rhACE2 were similar to levels in untreated controls (Figure 5).

### 2.5. Exposure of HLMEC and Neutrophils to S-Proteins and Endothelial–Neutrophil Interactions Inhibits TFPI

**Figure 6:**
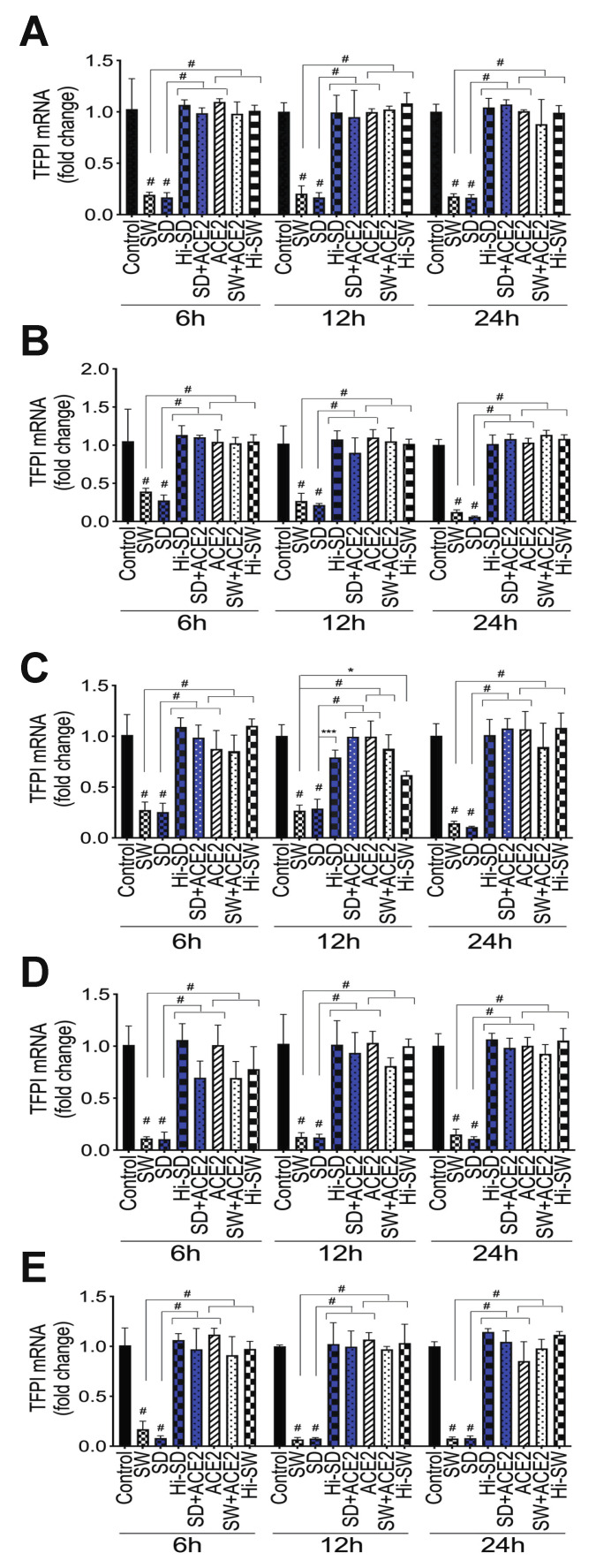
S-proteins and endothelial–neutrophil interactions inhibit TFPI transcription in HLMEC and neutrophils. (**A**) HLMEC treated with S-proteins (1 nM SW or SD) for 6–24 h; (**B**,**C**) HLMEC treated 6 h with S-proteins, washed, and co-cultured with neutrophils for 6–24 h; (**D**,**E**) Human neutrophils treated 6 h with S-proteins, washed, and co-cultured with HLMEC for 6–24 h. Following treatment, each cell type was harvested separately and TFPI mRNA levels in endothelial cells (**A**,**B**,**D**) and neutrophils (**C**,**E**) were quantified by real-time PCR. Data presented as mean ± standard deviation. Control: untreated cells; ACE2: cells treated with rhACE2 (1 µg/mL); Hi: cells treated with heat-inactivated S-proteins. * *p* = 0.025; *** *p* = 0.0002; # *p* < 0.0001.

TFPI is the principal inhibitor of the coagulation cascade extrinsic pathway [31,32,33,34]. Exposure of HLMEC or neutrophils to S-proteins and endothelial–neutrophil interactions significantly downregulated TFPI transcription in both cell types. Compared to untreated cells, cells treated with Hi-SW, cells treated with Hi-SD, or cells pretreated with rhACE2, exposure of HLMEC to SW or SD (6–24 h) decreased TFPI mRNA by 5- to 6.5-fold (Figure 6A). Co-culture of SW- or SD-treated HLMEC with neutrophils decreased TFPI in HLMEC and neutrophils, respectively, by 2.6- to 17-fold (Figure 6B) and by 3- to 10-fold (Figure 6C). Co-culture of SW- or SD-treated neutrophils with HLMEC decreased TFPI in endothelial cells and neutrophils, respectively, by 6- to 10-fold (Figure 6D) and 5.36- to 15.7-fold (Figure 6E). rhACE2 blocked SW- and SD-induced downregulation of TFPI; TFPI levels in cells treated with Hi-SW, Hi-SD, or rhACE2 were similar to levels in untreated controls (Figure 6).

### 2.6. rTFPI Blocked S-Proteins-Induced Expression and Secretion of Factor-V, Thrombin, and Fibrinogen

**Figure 7:**
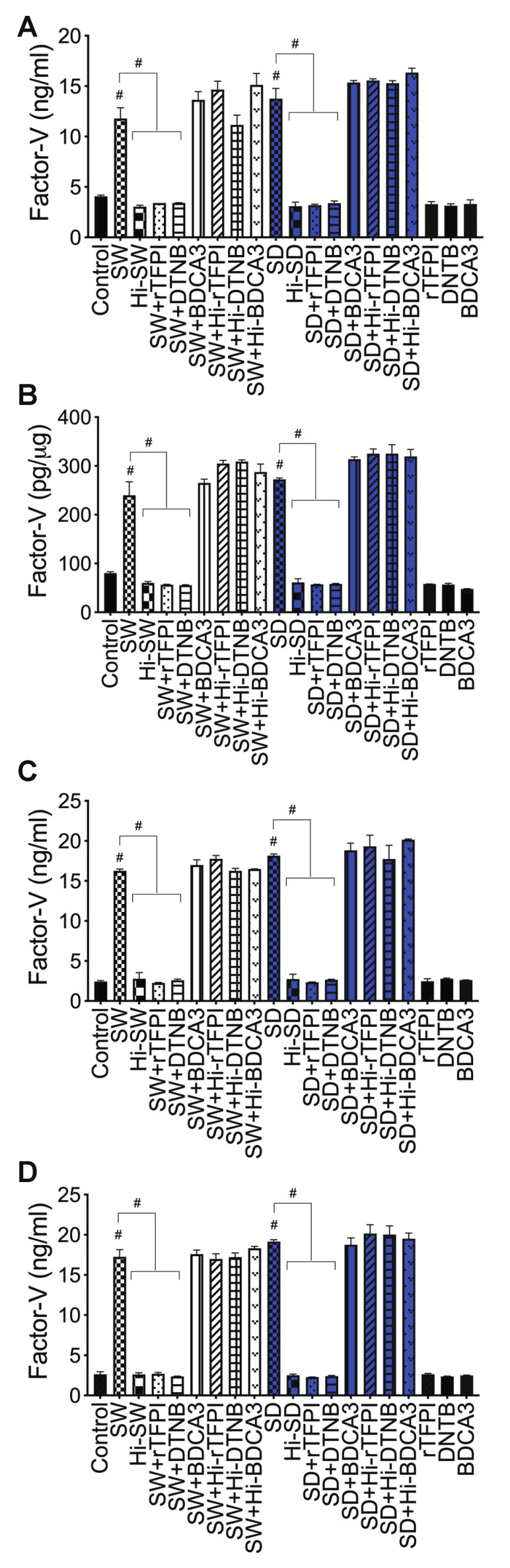
rTFPI and thiol blockers prevent S-protein-induced Factor-V expression and release in HLMEC, whereas TM had no effect: (**A**,**B**) HLMEC treated (24 h) with S-proteins (1 nM SW or SD), with or without rTFPI, DTNB, and BDCA3 (200 ng/mL); (**C**) HLMEC were treated 6 h with S-proteins, with or without rTFPI, DTNB, and BDCA3, washed, and co-cultured with neutrophils for 24 h; (**D**) human neutrophils were treated 6 h with S-proteins, with or without rTFPI, DTNB, and BDCA3, washed, and co-cultured with HLMEC for 24 h. Following treatments, Factor-V levels in culture supernatants (**A**,**C**,**D**) and endothelial cells lysates (**B**) were quantified by ELISA. Data presented as mean ± standard deviation. Control: untreated cells; Hi: heat-inactivated (SW, SD, rTFPI, DTNB, BDCA3). # *p* < 0.0001.

***Factor-V:*** Compared to untreated cells, cells treated with Hi-SW, or cells treated with Hi-SD, 24 h exposure of HLMEC to SW or SD increased Factor-V levels in culture supernatants (Figure 7A) and endothelial cell lysates (Figure 7B) by 3- to 4.5-fold. rTFPI blocked SW- and SD-induced Factor-V. Pretreatment with rTFPI reduced SW- and SD-induced Factor-V in HLMEC culture supernatants (by 3.5- to 4.3-fold, Figure 7A) and endothelial cell lysates (by 4.2- to 4.76-fold, Figure 7B). Co-culture of SW- or SD-treated HLMEC with neutrophils increased Factor-V levels in culture supernatants by 6- to 7.46-fold, and rTFPI blocked Factor-V expression and release: rTFPI reduced SW- and SD-induced Factor-V by 7.2- to 7.74-fold (Figure 7C). Co-culture of SW- or SD-treated neutrophils with HLMEC increased Factor-V levels in culture supernatants by 6.5- to 7.64-fold, and rTFPI blocked Factor-V expression and release: rTFPI reduced SW- and SD-induced Factor-V by 6.4- to 8.36-fold (Figure 7D). Hi-rTFPI had no effect on SW- or SD-induced Factor-V; Factor-V levels in cells exposed only to rTFPI were similar to levels in untreated controls (Figure 7). 

**Figure 8:**
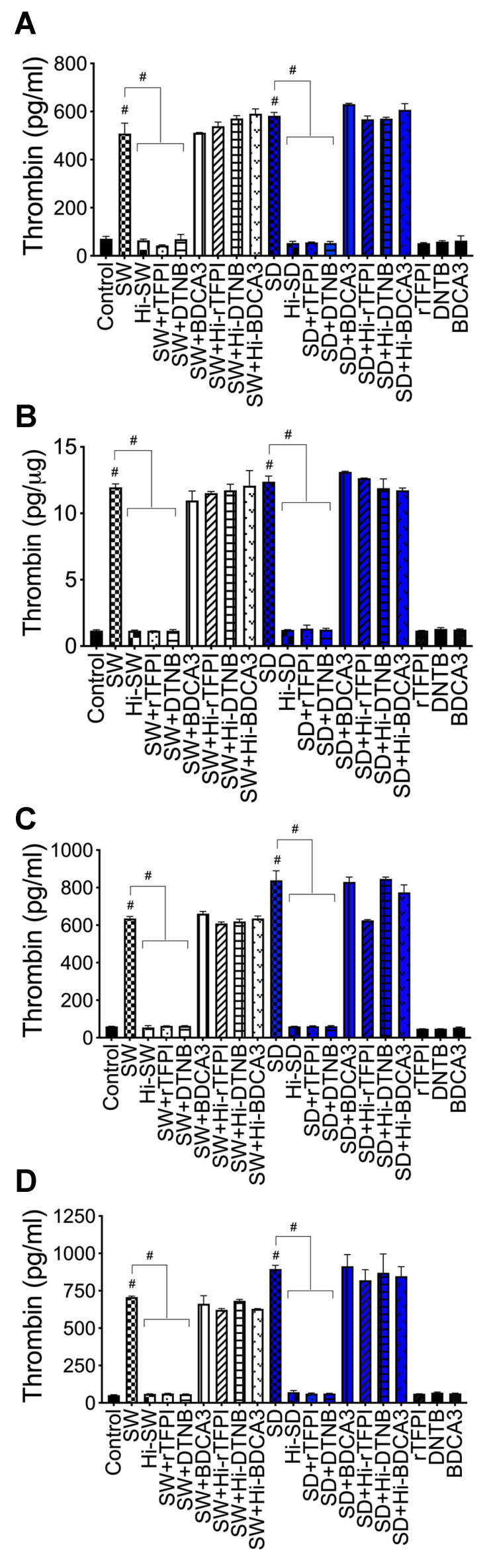
rTFPI and thiol blockers prevent S-proteins-induced thrombin expression and release in HLMEC, whereas TM had no effect: (**A**,**B**) HLMEC treated (24 h) with S-proteins (1 nM SW or SD), with or without rTFPI, DTNB, and BDCA3 (200 ng/mL); (**C**) HLMEC were treated 6 h with S-proteins, with or without rTFPI, DTNB, and BDCA3, washed, and co-cultured with neutrophils for 24 h; (**D**) human neutrophils were treated 6 h with S-proteins, with or without rTFPI, DTNB, and BDCA3, washed, and co-cultured with HLMEC for 24 h. Following treatments, thrombin levels in culture supernatants (**A**,**C**,**D**) and endothelial cells lysates (**B**) were quantified by ELISA. Data presented as mean ± standard deviation. Control: untreated cells; Hi: heat-inactivated (SW, SD, rTFPI, DTNB, BDCA3). # *p* < 0.0001.

***Thrombin***: Compared to untreated cells, cells treated with Hi-SW, or cells treated with Hi-SD, 24 h exposure of HLMEC to SW or SD increased thrombin levels in culture supernatants by 7- to 11-fold (Figure 8A) and in endothelial cell lysates by 10-fold (Figure 8B). rTFPI blocked SW- and SD-induced thrombin expression: rTFPI reduced SW- and SD-induced thrombin in HLMEC culture supernatants (by 10.5- to 11.74-fold, Figure 8A) and in endothelial cell lysates (by 9.37- to 10.36-fold, Figure 8B). Co-culture of SW- or SD-treated endothelial cells with neutrophils increased thrombin levels in culture supernatants by 10.5- to 14-fold, and rTFPI blocked thrombin expression and release: rTFPI reduced SW- and SD-induced thrombin by 10- to 13.6-fold (Figure 8C). Co-culture of SW- or SD-treated neutrophils with HLMEC increased thrombin levels in culture supernatants by 12- to 17-fold, and rTFPI blocked thrombin expression and release: rTFPI reduced SW- and SD-induced thrombin by 11.56- to 14.4-fold (Figure 8D). Hi-rTFPI had no effect on SW- or SD-induced thrombin; thrombin levels in cells exposed only to rTFPI were similar to levels in untreated controls (Figure 8).

**Figure 9:**
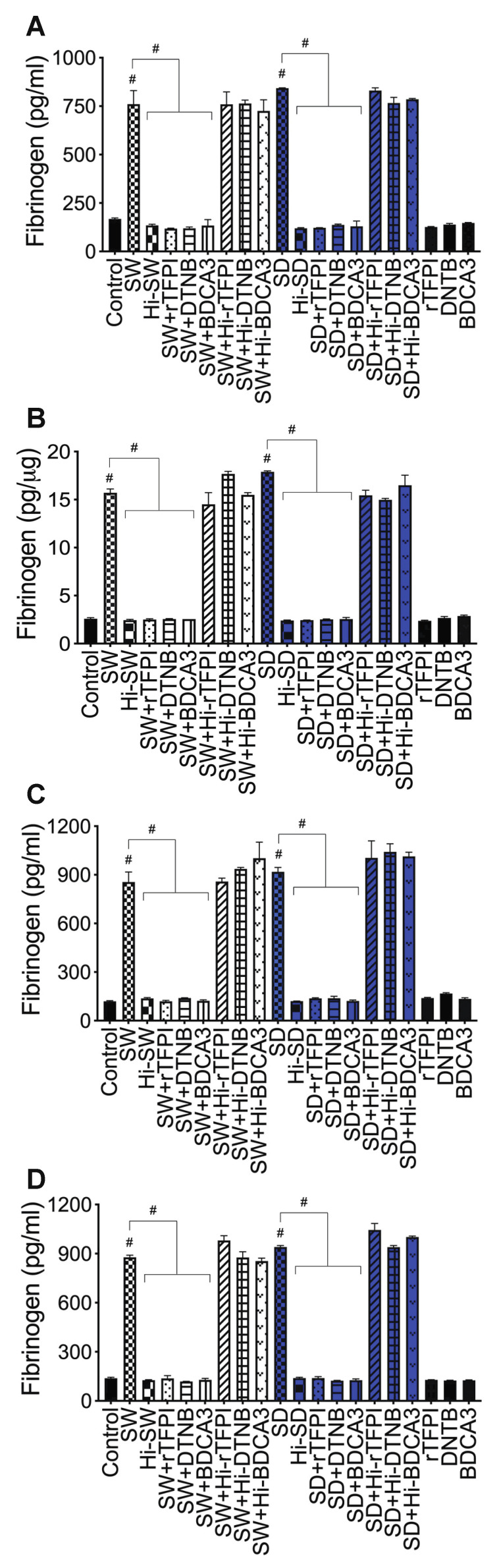
rTFPI, TM, and thiol blockers prevent S-proteins-induced fibrinogen expression and release in HLMEC: (**A**,**B**) HLMEC treated (24 h) with S-proteins (1 nM SW or SD), with or without rTFPI, DTNB, and BDCA3 (200 ng/mL); (**C**) HLMEC were treated 6 h with S-proteins, with or without rTFPI, DTNB, and BDCA3, washed, and co-cultured with neutrophils for 24 h; (**D**) human neutrophils were treated 6 h with S-proteins, with or without rTFPI, DTNB, and BDCA3, washed, and co-cultured with HLMEC for 24 h. Following treatments, fibrinogen levels in culture supernatants (**A**,**C**,**D**) and endothelial cell lysates (**B**) were quantified by ELISA. Data presented as mean ± standard deviation. Control: untreated cells; Hi: heat-inactivated (SW, SD, rTFPI, DTNB, BDCA3). # *p* < 0.0001.

***Fibrinogen***: Compared to untreated cells, cells treated with Hi-SW, or cells treated with Hi-SD, 24 h exposure of HLMEC to SW or SD increased fibrinogen levels in culture supernatants (Figure 9A) and endothelial cell lysates (Figure 9B) by 4.5- to 7.45-fold. rTFPI blocked SW- and SD-induced fibrinogen: rTFPI reduced SW- and SD-induced fibrinogen in HLMEC culture supernatants (by 6.4- to 7-fold, Figure 9A) and cell lysates (by 6.25- to 7.35-fold, Figure 9B). Co-culture of SW- or SD-treated endothelial cells with neutrophils increased fibrinogen levels in culture supernatants by 6- to 7.6-fold, and rTFPI blocked fibrinogen expression and release: rTFPI reduced SW- and SD-induced fibrinogen by 6.7- to 7.2-fold (Figure 9C). Co-culture of SW- or SD-treated neutrophils with HLMEC increased fibrinogen levels in culture supernatants by 6.3- to 7-fold and rTFPI blocked fibrinogen expression and release: rTFPI reduced SW- and SD-induced fibrinogen by 6.4 -to 6.73-fold (Figure 9D). Hi-rTFPI had no effect on SW- or SD-induced fibrinogen expression; fibrinogen levels in cells exposed only to rTFPI were similar to levels in untreated controls (Figure 9).

### 2.7. DTNB Blocked S-Proteins-Induced Expression and Secretion of Factor-V, Thrombin, and Fibrinogen

***Factor-V***: DTNB blocked SW- and SD-induced Factor-V in HLMEC, which reduced SW- and SD-induced Factor-V in HLMEC culture supernatants (Figure 7A) and endothelial cell lysates (Figure 7B) by 3.46- to 4.33-fold. DTNB blocked Factor-V induced by interaction of S-protein-treated HLMEC with neutrophils (Figure 7C) or interaction of S-protein-treated neutrophils with HLMEC (Figure 7D). DTNB reduced SW- and SD-induced Factor-V, respectively, by 6.27- to 6.85-fold (Figure 7C) and by 7.27- to 8-fold (Figure 7D). Hi-DTNB had no effect on SW- or SD-induced Factor-V, and Factor-V levels in cells exposed only to DTNB were similar to levels in untreated controls (Figure 7).

***Thrombin***: DTNB blocked SW- and SD-induced thrombin expression and secretion in HLMEC, which reduced SW- and SD-induced thrombin in HLMEC culture supernatants (Figure 8A) and endothelial cell lysates (Figure 8B) by 7.34- to 11-fold. DTNB blocked the increased thrombin induced by interaction of S-protein-treated HLMEC with neutrophils (Figure 8C) or interaction of S-protein-treated neutrophils with HLMEC (Figure 8D). DTNB reduced SW- and SD-induced thrombin, respectively, by 12- to 14.3-fold (Figure 8C), and by 10- to 13.6-fold (Figure 8D). Hi-DTNB had no effect on SW- or SD-induced thrombin expression, and thrombin levels in cells exposed only to DTNB were similar to levels in untreated controls (Figure 8).

***Fibrinogen***: DTNB blocked SW- and SD-induced fibrinogen expression and secretion in HLMEC, which reduced SW- and SD-induced fibrinogen in HLMEC culture supernatants (Figure 9A) and endothelial cell lysates (Figure 9B) by 6- to 7-fold. DTNB blocked the increased fibrinogen induced by interaction of S-protein-treated HLMEC with neutrophils (Figure 9C) or interaction of S-protein-treated neutrophils with HLMEC (Figure 9D). DTNB reduced SW- and SD-induced fibrinogen expression by 6- to 7.6-fold (Figure 9C,D). Hi-DTNB had no effect on SW- or SD-induced fibrinogen, and fibrinogen levels in cells exposed only to DTNB were similar to levels in untreated controls (Figure 9).

### 2.8. Thrombomodulin Blocked S-Protein-Induced Expression and Secretion of Fibrinogen but Had No Effect on S-Protein-Induced Expression and Secretion of Factor-V or Thrombin

***Fibrinogen***: Thrombomodulin (TM, BDCA3) blocked SW- and SD-induced fibrinogen expression and secretion in HLMEC, which reduced SW- and SD-induced fibrinogen in HLMEC culture supernatants (Figure 9A) and endothelial cell lysates (Figure 9B) by 5- to 7-fold. BDCA3 blocked the increased fibrinogen induced by interaction of S-protein-treated HLMEC with neutrophils (Figure 9C) or interaction of S-protein-treated neutrophils with HLMEC (Figure 9D). BDCA3 reduced SW- and SD-induced fibrinogen expression and secretion by 6.8- to 7.54-fold (Figure 9C,D). Hi-BDCA3 had no effect on SW- or SD-induced fibrinogen, and fibrinogen levels in cells exposed only to BDCA3 were similar to levels in untreated controls (Figure 9). 

***Factor-V and thrombin***: BDCA3 had no effect on SW- or SD-induced Factor-V (Figure 7) or thrombin (Figure 8) expression.

## 3. Discussion

COVID-19 progression is characterized by acute respiratory distress syndrome associated with increased lung injury, coagulopathy, and thrombosis of the alveolar capillaries [6,7,8,9]. The pathogenesis of these pulmonary complications in COVID-19 patients is not well-known. SARS-CoV-2 infect target cells by binding its S-protein to cellular ACE2, and human microvascular endothelial cells [18,19,20] and neutrophils [16,17] express ACE2. In the present study, we demonstrate that exposure of primary HLMEC to SARS-CoV-2 S-proteins induces transcriptional upregulation of TF and significantly increases the expression and secretion of Factor-V, thrombin, and fibrinogen, all of which are clotting factors known to activate the coagulation cascade and induce thromboinflammation [28,29,30]. We further demonstrate that co-culture of human neutrophils with S-protein-exposed HLMEC or co-culture of HLMEC with S-protein-exposed neutrophils significantly increased TF, Factor-V, thrombin, and fibrinogen in both cell populations. S-proteins effects were further demonstrated by control experiments, showing that similar concentrations of heat-inactivated S-proteins had no effect and that using rhACE2 to prevent S-protein binding to cellular ACE2 abrogated S-protein-induced TF, Factor-V, thrombin, and fibrinogen.

**Figure 10:**
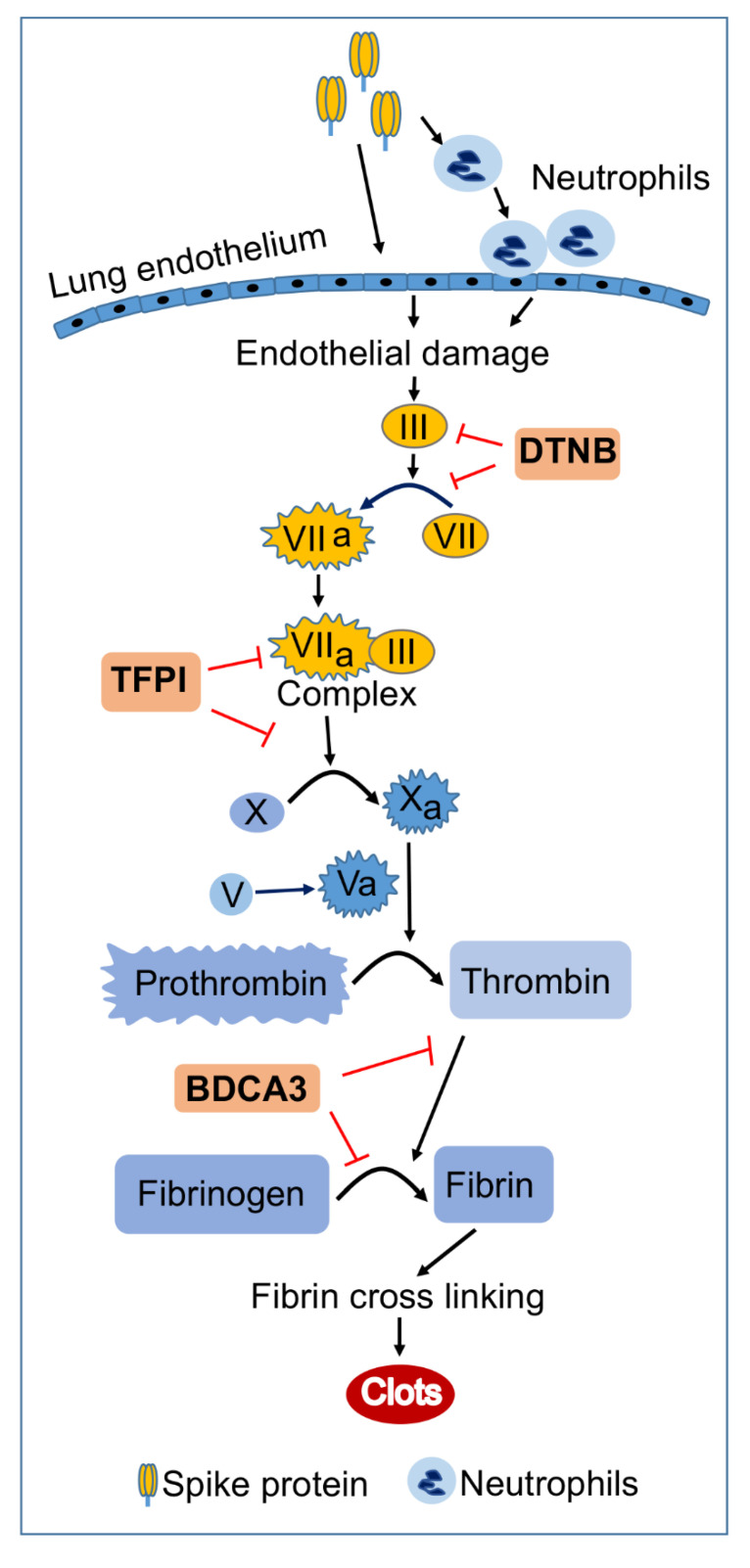
Model illustrating the coagulation cascade pathway in S-protein-induced TF, Factor-V, thrombin, and fibrinogen. Arrows indicate direct activation. The red ⊥ symbol indicate pharmacological inhibitors. Yellow boxes indicate factors associated with the extrinsic pathway of blood coagulation. Blue boxes indicate factors associated with the common pathway of blood coagulation.

These results corroborate clinical findings in humans and animals’ studies. Study of COVID-19 patients, as well as *in vitro* and *in vivo* studies, showed that S-proteins induced ACE2-mediated endothelial activation, endothelial injury, oxidative stress, and inflammation, associated with increased platelets aggregation [21,23,39]. Studies of SARS-CoV-2 infected patients showed increased TF in the plasma and cutaneous vessels associated with microthrombi, with the highest TF levels associated with severe COVID-19 [24,40,41,42,43]. TF is principally produced by vascular endothelial and perivascular cells but can also be produced by leukocytes [29,44,45,46]. TF is the effector that triggers the coagulation cascade extrinsic pathway. In this pathway, TF produced activates Factor-VII (VIIa), resulting in a TF/VIIa complex that further activates Factor-X. Activated Factor-X converts prothrombin to thrombin, and this thrombin production is catalyzed by activated Factor-V [28,29,30] (Figure 10). Thrombin catalyzes the formation of fibrin clots; it cleaves fibrinogen to generate fibrin fibers that aggregate and crosslink to form fibrin clots [37,38] (Figure 10). There is evidence of increased plasma thrombin, high thrombin activity, and increased fibrinogen in COVID-19 patients, with the highest levels observed in severely ill patients [47]. Autopsy study of these patients showed severe lung injury characterized by extensive damage to lung endothelial cells, thrombosis of alveolar capillaries, inflammation, and recruitment of neutrophils/leukocytes to sites of inflamed endothelium [6,7,8,9,48]. Our current data suggests that SARS-CoV-2 S-proteins play a major role in the endotheliopathy and coagulopathy observed in COVID-19. In fact, post-mortem studies of COVID-19 patients showed that in addition to endothelial apoptosis and microvascular thrombosis, there were viral particles and S-proteins within microvessels, endothelial cells, and activated neutrophils/leukocytes sequestrated within the vascular endothelium [7,12,23,49]. S-protein-induced thrombin can further accentuate/propagate the coagulation cascade in a Factor-XI feedback activation loop [50]. Thrombin can also activate endothelial cells and induce proteolytic activation of other factors that drive the coagulation cascade, resulting in increased coagulopathy, inflammation, and thrombosis [50,51,52].

Since the beginning of the COVID-19 pandemic, its disease epidemiology has been characterized by waves of infections driven by SARS-CoV-2 genetic variants and subvariants [53,54,55,56]. Whether or not such genetic variations affect disease pathogenesis remains to be determined. Our data show that compared to SW, exposure of HLMEC or neutrophils to SD and endothelial–neutrophil interactions induced significantly higher TF levels. This suggests that SARS-CoV-2 variants and genotype can influence the production of pro-thrombotic factors and disease pathology in infected individuals. We demonstrate that S-protein-induced upregulation of TF was associated with inhibition of TFPI, a natural anti-coagulant and principal regulator of the coagulation cascade extrinsic pathway. TFPI is a serine protease inhibitor that binds the VIIa/Xa complex to inhibits TF activity, blocking the coagulation cascade extrinsic pathway and preventing thrombosis [57,58,59]. Significantly, we demonstrate that rTFPI blocked S-protein-induced expression and secretion of Factor-V, thrombin, and fibrinogen. This suggests that therapeutic strategies against SARS-CoV-2-induced coagulopathy and thrombosis could include rTFPI supplementation.

In our current study, TM blocked the S-protein-induced expression and secretion of fibrinogen but had no effect on the S-protein-induced expression and secretion of Factor-V or thrombin. These findings agree with known coagulation pathways, since TM acts downstream of Factor-V and thrombin in the coagulation cascade and is not expected to affect upstream factors. TM is a natural anticoagulant that binds thrombin to form a stable thrombin–TM complex that induces fibrinolysis [35,36].

Our data demonstrates that DTNB blocked S-protein-induced expression and secretion of Factor-V, thrombin, and fibrinogen. DTNB is a thiol blocker; it reacts with free thiol groups to prevent the formation of disulfide bonds [60]. Formation of disulfide bonds is essential for TF activation and the increased TF activity that drives the coagulation signaling cascade extrinsic pathway [61,62,63]. For type-1 envelope viruses, such as coronaviruses and retroviruses, efficient viral fusion to the host’s cell membrane and viral entry into target cells depend on a balance between thiol and disulfide on the viral fusion proteins [64,65]. Changes in thiol/disulfide balance can trigger conformational changes that promote virus entry [66,67]. Removal of disulfide bonds on ACE2 and S-proteins can significantly impair S-protein RBD’s binding affinity for ACE2 [67], disrupt viral binding and prevent infection. In fact, DTNB has been shown to reduce the binding, entry, and infection of other envelope viruses into susceptible cells, including human immunodeficiency virus (HIV) [68], cytomegalovirus [69], and murine coronavirus [64]. Thiol-based drugs have also been shown to decrease the binding of SARS-CoV-2 S-proteins to ACE2, inhibit viral entry and infection, and significantly decrease lung neutrophilic inflammation [70,71]. The result of our current study suggests that exposure of HLMEC or neutrophils to S-proteins and endothelial–neutrophil interactions induce/increase TF and pro-thrombogenic factors via mechanisms involving/requiring free and functional thiol groups that likely participate in disulfide bonds formation during virus–cell interactions and binding. These findings further suggest that use of thiol-based drugs to cleave disulfide bridges could be a viable therapeutic strategy for preventing SARS-CoV-2-induced coagulopathy and thrombosis. Reducing oxidative stress could also have therapeutic benefits, as high doses of the antioxidant N-acetylcysteine have been shown to reduce COVID-19 mortality [72].

In summary, our current study demonstrates that exposure of primary HLMEC or neutrophils to S-proteins and endothelial–neutrophil interactions induced pro-coagulant and thrombogenic factors. This was associated with inhibition of TFPI, and both rTFPI and a thiol blocker, DTNB, prevented S-protein-induced expression and secretion of Factor-V, thrombin, and fibrinogen. These results suggests that following SARS-CoV-2 contact with the pulmonary endothelium or neutrophils and endothelial–neutrophil interactions, viral S-proteins induce coagulopathy via the TF pathway and mechanisms involving free and functional thiol groups. These findings also suggest that therapeutic strategies against SARS-CoV-2-induced coagulopathy and thrombosis could include rTFPI supplementation and/or thiol-based drugs.

## 4. Materials and Methods

### 4.1. Reagents

Recombinant SARS-CoV-2 S-proteins, SW (cat. #10549-CV-100), SD (cat. #10878-CV-100), rhACE2 (cat. #933-ZN-010), rTFPI (cat. #2974-PI-010), and TM (BDCA3, cat. #3947-PA-010) were purchased from R&D Systems (Minneapolis, MN, USA). DTNB (cat. #D218200) was from Sigma-Aldrich (St. Louis, MO, USA). Anti-human CD66b (cat. #60086AZ) and anti-human CD45 (cat. #600-18PE) antibodies were from Stemcell technologies (Cambridge, MA, USA); anti-human CD16 antibody (cat. #165-040) was from Ancell Corporation (Stillwater, MN, USA).

### 4.2. Cytotoxicity Assay

Cytotoxicity of S-proteins, rTFPI, DTNB, and BDAC3 was assessed by AlamarBlue assay, using the AlamarBlue reagent (cat# BUF012, Bio-Rad, Hercules, CA, USA), in accordance with the manufacturer’s instructions. Doses shown not to decrease cell viability (Figure 1) were used for subsequent experiments. For this study, S-proteins (both SW and SD) were used at 1 nM and rTFPI, DTNB, and BDCA3 at 200 ng/mL, based on viability tests (Figure 1) and previous studies [73].

### 4.3. Primary Human Lung Microvascular Endothelial Cells and Neutrophils

Primary HLMEC (cat. #CC-2527) was obtained from Lonza (Houston, TX, USA) and cultured to confluence in GM-2 endothelial media supplemented with growth factors (Lonza cat. #CC-4147), as we previously described [74,75,76]. Cells at passages 2–4 were used in this study. Blood samples was obtained from HIV-1, HIV-2, and hepatitis-B seronegative donors, as we previously described [76,77]. Human neutrophils were isolated from fresh donor’s blood using EasySep direct human neutrophil isolation kit (cat. #19666, Stemcell Technologies), in accordance with the manufacturer’s protocol. The purity of isolated neutrophils was confirmed by FACS using antibodies to human CD16, CD66b, and CD45; and over 98% of isolated cells were pure neutrophils. Isolated neutrophils were transferred into RPMI 1640 medium supplemented with 10% fetal calf serum.

### 4.4. Cells Treatment and Endothelial-Neutrophil Co-Culture

For direct treatment with S-proteins, confluent HLMEC were exposed to 1 nM SW or SD and cultured for 6 h, 12 h, and 24 h. Cultures supernatants and cells were collected and used for ELISA and real-time PCR. For co-culture, confluent HLMEC in 24-well plates were treated with SW or SD (1 nM) for 6 h and washed three times with serum-free media to remove S-proteins. Neutrophils (7.5 × 10^5^ cells in 250 μL media) (1:3 endothelial-neutrophil ratio) were then added to each well; and endothelial and neutrophils were co-cultured for 6 h, 12 h, and 24 h. In separate experiments, purified human neutrophils were treated with SW or SD (1 nM) for 6 h, washed three times with serum-free media to remove S-proteins, and 7.5 × 10^5^ neutrophils (in 250 μL media) added to HLMEC (in 24-well plates); then, both cell types were co-cultured for 6 h, 12 h, and 24 h. Controls included untreated cells, cells treated with heat-inactivated (Hi) S-proteins, and cells pretreated with rhACE2 (1 μg/mL) to block S-protein binding. Additional experimental conditions included cells pretreated with rTFPI, DTNB, or BDCA3 (200 ng/mL). Following co-culture, supernatants were collected, and neutrophils and HLMEC were harvested separately. Media containing neutrophils suspension were collected into 15-mL tubes; adherent HLMEC were then rinsed three times with phosphate buffered saline (500 μL/well) to collect any residual neutrophils. Neutrophils were pelleted by centrifugation (226× *g*, 5 min). Adherent HLMEC were again washed with phosphate buffered saline and harvested by trypsinization and centrifugation (226× *g*, 5 min). HLMEC and neutrophils were separately used for ELISA and real-time PCR.

### 4.5. RNA Isolation and Real-Time PCR

Total RNA was extracted from cells using the Trizol reagent (Life Technologies-Ambion, Austin, TX, USA), in accordance with the manufacturer’s protocol. RNA yield and quality were checked using a NanoDrop spectrophotometer (NanoDrop Technologies, Wilmington, DE, USA), and, for all samples, 260/280 absorbance ratios were ≥2. Reverse transcription was performed using Verso cDNA synthesis kit (ThermoFisher); 1 µg RNA in 11 µL nuclease-free water was mixed with 4 µL of 5X cDNA synthesis buffer, 2 µL dNTP mix, 1 µL random hexamers, 1 µL reverse transcriptase enhancer, and 1 µL Verso enzyme mix. Amplification conditions were as follows: 1 cycle of 42 °C for 30 min and 95 °C for 2 min.

Quantitative real-time PCR was performed using the 384-well block of a LightCycler 480 II (Roche) Real-Time PCR System. For each reaction, 500 ng cDNA in 5 µL nuclease-free water was mixed with 3 µL PCR grade water, 10 µL of 2X LightCycler 480 Probe master mix, and 1 µL of 20X TaqMan primer-probe mix. Cycling conditions were as follows: 95 °C, 5 min with a ramp rate of 4.8 °C/s; followed by 45 cycles of 95 °C, 10 s, 4.8 °C/s; 60 °C, 15 s, 2.5 °C/s; and 72 °C, 1 s, 4.8 °C/s; and held at 40 °C, 10 s, 2 °C/s. Factor-III and TFPI mRNA levels were quantified using the Delta-CT method, in accordance with the instruction of the LightCycler 480 manufacturer’s software manual and normalized to the sample’s GAPDH levels. All primers were obtained from Applied Biosystems (Waltham, MA, USA), and primers’ IDs were as follows: Factor-III (Hs01076029_m1), TFPI (Hs00409210_m1), and GAPDH (Hs02786624_g1).

### 4.6. Human Factor-V, Thrombin, and Fibrinogen ELISA

Following treatments, culture supernatants and cells were collected, as described above. Cells were lysed in mammalian cell lysis buffer (CelLytic M, cat. #C2978, Sigma), and their protein content was quantified using the bicinchoninic acid assay, as previously described [78,79,80]. Factor-V, thrombin, and fibrinogen levels in each culture supernatant (100 μL) and cell lysates (100 μL containing 50 μg protein) were quantified by ELISA using human Factor-V (cat. #ab137976), thrombin (cat. #ab270210), and fibrinogen (cat. #ab241383) ELISA kits (from Abcam), in accordance with the manufacturer’s protocols. Standard curves from human Factor-V, thrombin, and fibrinogen reference standards (provided with each kit) were used, respectively, to quantify Factor-V, thrombin, and fibrinogen (activated and non-activated forms) levels in each supernatant and cell lysates sample.

### 4.7. Statistical Analysis

Data were analyzed by Student’s t-test (two-tailed) or by one- or two-way analysis of variance followed by Tukey’s multiple-comparisons tests using GraphPad Prism 9.0 (GraphPad Software, La Jolla, CA, USA). Data are presented as mean ± standard deviation, and the threshold of significance level was 0.05.

## Data Availability

All data generated or analyzed during this study are included in this publication and/or are available from the corresponding author on reasonable request.

## Abbreviations

DTNB	5:5′-dithio-bis-(2-nitrobenzoic acid
TFPI	Tissue factor pathway inhibitor
rTFPI	Recombinant tissue factor pathway inhibitor
BDCA3/TM	Thrombomodulin
III/TF	Factor-III/tissue factor
VII	Factor-VII
VIIa	Activated factor-VII
X	Factor-X
Xa	Activated factor-X
V	Factor-V
Va	Activated factor-V
SARS-CoV-2	Severe acute respiratory syndrome coronavirus-2
COVID-19	Coronavirus disease 2019
ACE2	Angiotensin-converting enzyme-2
rhACE2	Recombinant human ACE2
S-proteins	Spike proteins
SW	Spike protein: Wuhan variant
SD	Spike protein: Delta variant
Hi	Heat-inactivated
RBD	Receptor binding domain
PD	Protease domain
ELISA	Enzyme-linked immunosorbent assay
PCR	Polymerase chain reaction
cDNA	Complementary DNA
GAPDH	Glyceraldehyde-3-phosphate dehydrogenase
Cat. #	Catalog number
